# Supplementary material for: Comparative analysis of neuronal proteolytic pathways reveals neuron-specific and sub-compartmental-specific capacities with aging
Source: Cell Mol Life Sci. 2026 Aug 13;83(1):308. doi: 10.1007/s00018-026-06383-y (PMC13481654; doi:10.1007/s00018-026-06383-y)
Supplement: Supplementary file 1 [file 18_2026_6383_MOESM2_ESM.docx]

**Supplementary Information**

**Figure S1** Depth and consistency of proteome coverage across ages and compartments in mouse and *C. elegans* datasets. **A)** Total number of quantified protein groups identified in mouse cortical synaptosome preparations from 2‑, 8‑, and 18‑month‑old animals across all biological replicates, showing comparable depth of coverage between age groups. **B)** Total number of quantified protein groups in *C. elegans* Day 4 and Day 7 samples (N = 3; ~6000 nematodes per experiment) for whole neurons (GFP^+^/mCherry^+^), synaptic compartments (GFP^+^), and neuronal cell bodies (mCherry^+^), demonstrating similar proteome depth between ages and compartments. **C)** Total number of quantified protein groups in *C. elegans* Day 4 and Day 10 samples (N = 3; ~6000 nematodes per experiment) for the same three neuronal fractions, again indicating stable identification numbers across ages and compartments.

**Figure S2** Gene Ontology Biological Process enrichment across aging mouse cortical synaptosomes. Differentially abundant proteins (>1.5‑fold change, Q < 0.05) were analyzed with Enrichr using the GO Biological Process ontology. Bar plots display significantly enriched terms (adjusted Q < 0.05) for pairwise comparisons between 2‑, 8‑, and 18‑month‑old cortical synaptosome proteomes: **A)** terms enriched in 2‑ versus 18‑month synaptosomes, **B)** terms depleted in 2‑ versus 18‑month synaptosomes, **C)** terms enriched in 2‑ versus 8‑month synaptosomes, **D)** terms depleted in 2‑ versus 8‑month synaptosomes. Bars represent the Enrichr combined enrichment score (−log P value multiplied by the odds ratio); the higher values indicate stronger over‑representation of the corresponding GO term in the indicated comparison.

**Figure S3** Gene Ontology Biological Process enrichment across aging mouse cortical synaptosomes. Differentially abundant proteins (>1.5‑fold change, Q < 0.05) were analyzed with Enrichr using the GO Biological Process ontology. Bar plots display significantly enriched terms (adjusted Q < 0.05) for pairwise comparisons between 8‑ and 18‑month‑old cortical synaptosome proteomes. GO analysis for the depleted candidates did not have significant (Q<0.05) hits across GO data bases (due to low number of candidate hits) and hence not shown. Bars represent the Enrichr combined enrichment score (−log P value multiplied by the odds ratio); the higher values indicate stronger over‑representation of the corresponding GO term.

**Figure S4** Gene Ontology Biological Process enrichment across aging *C. elegans* synapses. Differentially abundant proteins (>2‑fold change, P < 0.01) were analyzed with Enrichr using the GO Biological Process ontology. Bar plots display significantly enriched terms (adjusted Q < 0.05) for pairwise comparisons between 4‑, 7‑, and 10‑day‑old synapse proteomes: **A)** terms enriched in 4‑ versus 7‑day old synapses, **B)** terms depleted in 4‑ versus 7‑day old synapses, **C)** terms enriched in 4‑ versus 10‑day old synapses, **D)** terms depleted in 4‑ versus 10‑day old synapses. Bars represent the Enrichr combined enrichment score (−log P value multiplied by the odds ratio); the higher values indicate stronger over‑representation of the corresponding GO term in the indicated comparison.

**Figure S5** Sunburst representations of GO enrichment for synaptic proteins during aging in *C. elegans*. Differentially abundant proteins (>2‑fold change, P < 0.01) from day‑4 versus day‑7 and day‑4 versus day‑10 synaptic proteomes were analyzed using the SynGO analysis tool by Gene Ontology Biological Process and Cellular Component categories. **A)** Sunburst map of Biological Process terms enriched in day‑4 versus day‑7 synapses. **B)** Corresponding Cellular Component map for enriched proteins in day‑4 versus day‑7 synapses. **C)** Sunburst map of Biological Process terms enriched in day‑4 versus day‑10 synapses. **D)** Cellular Component map for enriched proteins in day‑4 versus day‑10 synapses. Segment color encodes the number of proteins annotated to each GO term (gene count), with warmer colors indicating higher counts.

**Figure S6** Raw representative images of western blot membranes probing the 20S alpha subunits of the proteasome. Anti-Tubulin is used as a control. The membranes are from one trial (out of 2) of samples from cortical cytoplasmic fraction (upper left), cortical synaptosomes (upper right), cerebellar cytoplasmic fraction (lower left) and cerebellar synaptosomes (lower right). For each membrane, samples from 4 animals per age group (2 months old, 8 months old and 18 months old) are loaded consecutively. A protein ladder is loaded on the far left and far right of each membrane, with the molecular weights indicated in kDa.

**Figure S7** Scatter plots depicting the average number of autophagosomes (in yellow) and autolysosomes (in red) counted at the dorsal nerve cord of day 4 and day 7 old nematodes treated with chloroquine (20 mM) or ddH_2_O as control. Experiments were carried out in triplicates, and each data point represents one animal. Significance was assessed with an ordinary two way ANOVA with Tukey’s multiple comparison post hoc test (p > 0.05 (ns), p < 0.05 (*), p < 0.01 (**), p < 0.001 (***) and p < 0.0001 (****)).

**Table S.1 Synaptic versus somatic candidate proteins with significant differential expression between subcompartments.**

Synaptic versus somatic candidate proteins with significant age-dependent differential expression in *C. elegans*. Listed are proteins that are significantly enriched (A) or depleted (B) in the synaptic fraction relative to the somatic fraction at day 4, compared with day 7 animals (Q < 0.05, fold change > 1.5). Protein ID, description, average log_2_ ratio (synapse/soma), and adjusted Q value are shown for each candidate.

A. **Enriched in Day 4 and depleted in Day 7**

| **Protein ID** | **Protein Description** | **AVG.Log2.Ratio** | **Q value** |
| --- | --- | --- | --- |
| O44145 | PERMeable eggshell | 4.22 | 3.3E-02 |
| SUN1 | Sun domain-containing protein 1 | 3.53 | 4.6E-03 |
| RM35 | Probable 39S ribosomal protein L35, mitochondrial | 3.48 | 2.7E-03 |
| SNG1 | Synaptogyrin homolog 1 | 3.15 | 5.7E-03 |
| UNC17 | Vesicular acetylcholine transporter unc-17 | 2.80 | 2.9E-05 |
| RL5 | 60S ribosomal protein L5 | 2.51 | 3.0E-07 |
| G5EGI7 | 2 (Zwei) IG domain protein | 2.49 | 9.4E-03 |
| SYT1 | Synaptotagmin-1;SyNapTotagmin | 2.48 | 6.5E-16 |
| Q21925 | ProstaGlandin E Synthase homolog | 2.40 | 9.0E-09 |
| CAN5 | Calpain-5 | 2.32 | 4.6E-02 |
| RL31 | 60S ribosomal protein L31 | 2.14 | 5.0E-04 |
| NDUB7 | NADH dehydrogenase [ubiquinone] 1 beta subcomplex subunit 7 | 2.09 | 3.5E-02 |
| SC5A7 | High-affinity choline transporter 1 | 2.04 | 1.5E-04 |
| RL6 | 60S ribosomal protein L6 | 2.03 | 1.4E-07 |
| DYL1 | Dynein light chain 1, cytoplasmic | 1.96 | 4.4E-02 |
| Q86S81 | ELRR (Extracellular Leucine-Rich Repeat) ONly | 1.92 | 3.5E-02 |
| Q86NE0 | ASpartyl Protease | 1.91 | 2.4E-02 |
| O16619 | DeHydrogenases, Short chain | 1.86 | 4.3E-10 |
| EAA1 | Excitatory amino acid transporter | 1.74 | 4.4E-02 |
| UNC9 | Innexin unc-9 | 1.74 | 2.0E-03 |
| CATA1 | Peroxisomal catalase 1 | 1.73 | 1.0E-03 |
| Q9XWE1 | DNaJ domain (Prokaryotic heat shock protein) | 1.72 | 1.3E-03 |
| APOP1 | APOPT family protein Y39B6A.34, mitochondrial | 1.70 | 1.7E-02 |
| Q9GYJ9 | Sorting NeXin | 1.69 | 2.8E-02 |
| Q95YD8 | Isocitrate dehydrogenase [NAD] subunit, mitochondrial | 1.69 | 9.2E-06 |
| RL15 | 60S ribosomal protein L15 | 1.67 | 6.2E-04 |
| Q20992 | Acyl-coenzyme A oxidase | 1.67 | 1.8E-03 |
| Q22800 | NADH Ubiquinone oxidoreductase Fe-S protein | 1.67 | 3.0E-05 |
| H2KZ88 | UDP-GlucuronosylTransferase | 1.64 | 1.9E-04 |
| GLYC | Serine hydroxymethyltransferase | 1.61 | 8.6E-05 |
| PISD | Phosphatidylserine decarboxylase proenzyme, mitochondrial | 1.57 | 4.6E-02 |
| P91493 | SyNapTotagmin | 1.56 | 1.2E-03 |
| SUMO | Small ubiquitin-related modifier | 1.55 | 5.0E-05 |
| Q9U3Q0 | Mitochondrial Ribosomal Protein, Small | 1.55 | 5.2E-03 |
| CHSSB | Chondroitin sulfate synthase mig-22 | 1.54 | 2.1E-02 |
| SUV3 | ATP-dependent RNA helicase SUV3 homolog, mitochondrial | 1.54 | 3.4E-03 |
| O16521 | NADH-cytochrome b5 reductase | 1.54 | 3.3E-02 |
| O01869 | Ribosomal Protein, Small subunit | 1.53 | 1.7E-06 |
| Q9N4P2 | GLutaRedoXin | 1.51 | 4.7E-03 |
| Q95YB2 | Acyl CoA DeHydrogenase | 1.51 | 7.1E-11 |
| Q21083 | Mitochondrial Ribosomal Protein, Large | 1.51 | 8.2E-10 |
| OXDD2 | D-aspartate oxidase 2 | 1.50 | 2.5E-05 |
| Q93896 | MonoCarboxylate Transporter family | 1.50 | 5.6E-04 |
| O16922 | UDP-GlucuronosylTransferase | 1.47 | 8.9E-03 |
| A5PEY3 | DNA-directed RNA polymerase | 1.45 | 1.2E-03 |
| O17694 | RNAi-Induced Longevity | 1.43 | 4.9E-02 |
| RT24 | 28S ribosomal protein S24, mitochondrial | 1.41 | 4.2E-04 |
| Q21154 | MICOS complex subunit | 1.41 | 2.9E-03 |
| A0A0K3ARZ7 | F-box A protein | 1.40 | 3.7E-02 |
| STX1A | Syntaxin-1A homolog | 1.40 | 2.9E-03 |
| KMO | Kynurenine 3-monooxygenase | 1.40 | 1.1E-07 |
| G5EEG6 | Carrier protein (C2) | 1.40 | 2.1E-03 |
| Q95R12 | ACyLtransferase-like | 1.40 | 1.5E-03 |
| Q95X39 | Mitochondrial Ribosomal Protein, Large | 1.39 | 1.2E-03 |
| NNRD | ATP-dependent (S)-NAD(P)H-hydrate dehydratase | 1.38 | 3.8E-02 |
| Q95PZ8 | Asparaginyl(N) Amino-acyl tRNA Synthetase | 1.38 | 1.3E-03 |
| O02147 | Carboxylic ester hydrolase | 1.36 | 4.3E-02 |
| ALH13 | Probable delta-1-pyrroline-5-carboxylate synthase | 1.35 | 1.2E-14 |
| DHTK1 | Probable 2-oxoglutarate dehydrogenase E1 component DHKTD1 homolog, mitochondrial | 1.35 | 6.5E-04 |
| Q9TZ90 | Mitochondrial Ribosomal Protein, Large | 1.35 | 2.9E-02 |
| O17643 | Isocitrate dehydrogenase [NADP] | 1.35 | 2.0E-03 |
| B2MZD0 | Vacuolar H ATPase;V-type proton ATPase subunit a | 1.34 | 1.9E-02 |
| Q18660 | Fatty Acid CoA Synthetase family | 1.34 | 5.0E-05 |
| Q22968 | Aminomethyltransferase | 1.33 | 3.2E-03 |
| I2HA98 | PhenylAlanine Hydroxylase;Probable phenylalanine-4-hydroxylase 1 | 1.29 | 8.2E-08 |
| G5EFC8 | Mammalian cell Death Associated Protein related | 1.28 | 4.3E-05 |
| Q23066 | Carnitine Palmitoyl Transferase | 1.27 | 4.6E-03 |
| G8JY83 | UBiQuitin;Polyubiquitin-A;Ubiquitin-60S ribosomal protein L40 | 1.27 | 1.2E-06 |
| G5EDB6 | ParaPleGiN AAA protease family | 1.27 | 1.9E-03 |
| AP2M | AP-2 complex subunit mu | 1.27 | 2.6E-05 |
| G5EG62 | UNC-45 | 1.26 | 2.9E-02 |
| Q23295 | Mitochondrial Processing Peptidase Beta | 1.26 | 1.8E-05 |
| ANI2 | Anillin-like protein 2 | 1.25 | 6.4E-03 |
| Q7KPW7 | Mitochondrial Ribosomal Protein, Large | 1.25 | 9.3E-06 |
| Q21021 | Nuclear Pore complex Protein | 1.24 | 4.6E-02 |
| Q17403 | UDP-GlucuronosylTransferase | 1.24 | 1.2E-02 |
| O01795 | DUal OXidase;Dual oxidase 1 | 1.24 | 2.4E-02 |
| CLPP1 | ATP-dependent Clp protease proteolytic subunit 1, mitochondrial | 1.24 | 2.2E-02 |
| ATPK | Putative ATP synthase subunit f, mitochondrial | 1.23 | 9.4E-04 |
| GSTK2 | Glutathione s-transferase kappa 2 | 1.22 | 2.7E-02 |
| D6R8W9 | RaBConnectin related | 1.21 | 1.9E-03 |
| Q20600 | TBC (Tre-2/Bub2/Cdc16) domain family | 1.21 | 2.1E-02 |
| RT05 | Putative 28S ribosomal protein S5, mitochondrial | 1.21 | 6.0E-05 |
| LACB2 | Beta-lactamase-like protein 2 homolog | 1.19 | 1.3E-03 |
| RS16 | 40S ribosomal protein S16 | 1.19 | 6.2E-06 |
| D9N122 | Eps15 (Endocytosis protein) Homologous Sequence;EHS-1 | 1.18 | 6.7E-03 |
| NDUA5 | Probable NADH dehydrogenase [ubiquinone] 1 alpha subcomplex subunit 5 | 1.18 | 1.1E-02 |
| SIP1 | Stress-induced protein 1 | 1.18 | 2.7E-03 |
| AT1B1 | Sodium/potassium-transporting ATPase subunit beta-1 | 1.18 | 4.0E-05 |
| ATP5E | Putative ATP synthase subunit epsilon, mitochondrial | 1.18 | 1.5E-02 |
| GSK3 | Glycogen synthase kinase-3 | 1.17 | 1.3E-02 |
| Q8MXI1 | Ubiquinol-Cytochrome c oxidoReductase complex | 1.16 | 1.3E-02 |
| NDX7 | Putative nudix hydrolase 7 | 1.16 | 9.4E-04 |
| Q19813 | COLlagen | 1.14 | 4.8E-03 |
| CIA30 | Probable complex I intermediate-associated protein 30, mitochondrial | 1.14 | 3.0E-03 |
| RL13 | 60S ribosomal protein L13 | 1.13 | 3.9E-07 |
| O44727 | CalPoNin | 1.13 | 1.3E-03 |
| CRI3 | Conserved regulator of innate immunity protein 3 | 1.13 | 6.4E-03 |
| Q95XT5 | TRanslocon-Associated Protein | 1.13 | 3.6E-03 |
| COQ8 | Atypical kinase coq-8, mitochondrial | 1.12 | 7.4E-04 |
| UNC89 | Muscle M-line assembly protein unc-89 | 1.11 | 1.6E-02 |
| A9QY30 | Mitochondrial genome maintenance exonuclease 1 | 1.11 | 2.0E-03 |
| Q965T4 | Translational Activator of Cytochrome c Oxidase | 1.11 | 3.6E-05 |
| TIM50 | Mitochondrial import inner membrane translocase subunit TIM50 | 1.10 | 3.5E-06 |
| Q22470 | Mitochondrial Ribosomal Protein, Small | 1.09 | 8.3E-04 |
| MTX1 | Metaxin-1 homolog | 1.09 | 3.0E-02 |
| Q94056 | O-ACyltransferase homolog | 1.09 | 3.3E-02 |
| Q9XUB7 | Fatty Acid/Retinol binding protein | 1.08 | 2.1E-03 |
| ADF2 | Actin-depolymerizing factor 2, isoform c | 1.08 | 1.9E-03 |
| O18180 | Mitochondrial Ribosomal Protein, Large | 1.07 | 4.3E-04 |
| RM18 | 39S ribosomal protein L18, mitochondrial | 1.07 | 3.4E-02 |
| GPDM | Probable glycerol-3-phosphate dehydrogenase, mitochondrial | 1.07 | 2.9E-05 |
| GSTK1 | Glutathione S-transferase kappa 1 | 1.06 | 1.7E-02 |
| RS9 | 40S ribosomal protein S9 | 1.06 | 3.6E-06 |
| G5EFU2 | Adenine Nucleotide Translocator | 1.05 | 4.6E-03 |
| HGD | Homogentisate 1,2-dioxygenase | 1.05 | 4.8E-02 |
| EFGM | Elongation factor G, mitochondrial | 1.05 | 1.4E-09 |
| Q95XN2 | Mitochondrial Processing Peptidase Alpha | 1.05 | 5.5E-03 |
| Q20122 | Acyl carrier protein | 1.04 | 9.0E-04 |
| FABP3 | Fatty acid-binding protein homolog 3;Lipid Binding Protein | 1.04 | 3.2E-02 |
| Q95Y25 | Glutamyl-tRNA(Gln) amidotransferase subunit A, mitochondrial;Glutamyl-tRNA(Gln) amidotransferase subunit A, mitochondrial | 1.04 | 3.0E-02 |
| B1Q250 | UDP-glucuronosyltransferase | 1.04 | 1.7E-03 |
| Q7K742 | Daf-16-Dependent Longevity (WT but not daf-16 lifespan increased) | 1.04 | 1.9E-05 |
| Q6A575 | Mitochondrial Ribosomal Protein, Large | 1.04 | 1.7E-03 |
| COX5A | Cytochrome c oxidase subunit 5A, mitochondrial | 1.03 | 6.8E-04 |
| UNC18 | Putative acetylcholine regulator unc-18 | 1.02 | 2.4E-02 |
| SAM50 | SAM50-like protein gop-3 | 1.01 | 2.5E-03 |
| Q20203 | Calsequestrin | 1.01 | 2.0E-04 |
| DEOC | Putative deoxyribose-phosphate aldolase | 1.01 | 3.5E-02 |
| COX1 | Cytochrome c oxidase subunit 1 | 1.00 | 3.3E-06 |
| Q17447 | Fatty Acid Amide Hydrolase homolog | 1.00 | 2.7E-02 |
| Q95Y73 | Mitochondrial elongation factor G2 | 0.98 | 2.9E-02 |
| A0A0K3ATC0 | Tyramine Beta Hydroxylase;Tyramine beta-hydroxylase | 0.98 | 2.9E-02 |
| O17983 | ARRestin Domain protein | 0.98 | 1.1E-03 |
| Q9UA63 | Mitochondrial Ribosomal Protein, Large | 0.98 | 8.0E-05 |
| Q9NF11 | Hypoxanthine PhosphoRibosylTransferase homolog | 0.98 | 6.1E-04 |
| G5EBH7 | CALUmenin (Calcium-binding protein) homolog | 0.97 | 8.3E-03 |
| HCDH2 | Probable 3-hydroxyacyl-CoA dehydrogenase B0272.3 | 0.97 | 4.9E-03 |
| RT14 | Probable 40S ribosomal protein S14, mitochondrial | 0.96 | 3.6E-03 |
| Q9TZ33 | Ubiquinol-Cytochrome c oxidoReductase complex | 0.96 | 1.8E-05 |
| THILH | Acetyl-CoA acetyltransferase homolog, mitochondrial | 0.96 | 7.7E-05 |
| VPP1 | Probable V-type proton ATPase 116 kDa subunit a | 0.96 | 1.7E-08 |
| H2KYN3 | Acyl CoA DeHydrogenase | 0.96 | 2.5E-09 |
| H2KZF5 | Phosphoethanolamine MethylTransferase | 0.96 | 5.1E-05 |
| Q18697 | Phosphate transporter | 0.96 | 3.3E-02 |
| Q21569 | Beta-LACTamase domain containing | 0.95 | 4.3E-03 |
| ETFD | Electron transfer flavoprotein-ubiquinone oxidoreductase, mitochondrial | 0.95 | 1.6E-07 |
| FOLT2 | Folate-like transporter 2 | 0.95 | 3.8E-03 |
| TAM41 | Phosphatidate cytidylyltransferase, mitochondrial | 0.95 | 1.0E-04 |
| NLTP1 | Non-specific lipid-transfer protein-like 1 | 0.95 | 3.2E-02 |
| G4SRS5 | ABC Transporter, Mitochondrial | 0.95 | 2.4E-06 |
| Q9U1X0 | NADPH:adrenodoxin oxidoreductase, mitochondrial | 0.94 | 1.6E-02 |
| ACOX5 | Probable peroxisomal acyl-coenzyme A oxidase 5 | 0.94 | 6.1E-04 |
| SFXN5 | Sideroflexin-5 | 0.93 | 5.7E-04 |
| RT17 | 28S ribosomal protein S17, mitochondrial | 0.93 | 2.5E-02 |
| LONM | Lon protease homolog, mitochondrial | 0.93 | 1.0E-06 |
| MECR1 | Probable trans-2-enoyl-CoA reductase 1, mitochondrial | 0.93 | 1.2E-03 |
| O02207 | OXA mitochondrial inner membrane insertase homolog | 0.93 | 2.4E-05 |
| Q22251 | Cytochrome OXidase assembly protein | 0.92 | 1.1E-02 |
| UNC33 | Protein unc-33;Uncharacterized protein | 0.92 | 3.5E-03 |
| Q9BL86 | Mitochondrial Ribosomal Protein, Large | 0.91 | 6.0E-09 |
| SDHB | Succinate dehydrogenase [ubiquinone] iron-sulfur subunit, mitochondrial | 0.91 | 1.1E-04 |
| O44954 | Succinate dehydrogenase [ubiquinone] flavoprotein subunit, mitochondrial | 0.89 | 1.0E-07 |
| COQ5 | 2-methoxy-6-polyprenyl-1,4-benzoquinol methylase, mitochondrial | 0.88 | 3.5E-03 |
| G5ED08 | Stomatin-like protein UNC-1;Protein unc-1 | 0.88 | 2.8E-02 |
| Q9U2Q8 | Peptidyl-prolyl cis-trans isomerase | 0.88 | 1.3E-03 |
| DPYD | Dihydropyrimidine dehydrogenase [NADP(+)] | 0.87 | 1.3E-03 |
| MTX2 | Metaxin-2 homolog | 0.87 | 1.6E-03 |
| G5ECG7 | Defecation Suppressor of Clk-1 | 0.87 | 5.2E-05 |
| Q9NEI6 | Mitochondrial Ribosomal Protein, Small | 0.87 | 2.7E-02 |
| H2KYJ2 | TransThyretin-Related family domain | 0.86 | 5.9E-03 |
| RT23 | Probable 28S ribosomal protein S23, mitochondrial | 0.86 | 2.9E-03 |
| Q9GYI2 | Mitochondrial Ribosomal Protein, Small | 0.86 | 5.7E-03 |
| RM49 | Probable 39S ribosomal protein L49, mitochondrial | 0.85 | 1.3E-04 |
| G5EDJ3 | Equilibrative Nucleoside Transporter | 0.85 | 4.6E-03 |
| Q9U2S4 | ADP-Ribosylation Factor related | 0.84 | 8.5E-03 |
| Q8MPW0 | DEgenerin Like | 0.84 | 1.9E-05 |
| Q9XX18 | Mitochondrial Ribosomal Protein, Large | 0.84 | 2.3E-03 |
| Q9U295 | Carboxylic ester hydrolase | 0.84 | 1.7E-04 |
| Q18853 | CYtochrome C | 0.83 | 1.5E-05 |
| RT25 | Probable 28S ribosomal protein S25, mitochondrial | 0.83 | 3.6E-03 |
| G5ECI8 | Mitochondrial phenylalanyl-tRNA synthetase;Phenylalanyl Amino-acyl tRNA Synthetase | 0.83 | 1.9E-04 |
| O45148 | DihydroLipoamide S-SuccinylTransferase | 0.82 | 4.5E-02 |
| O44887 | Innexin | 0.82 | 5.8E-03 |
| LIAS | Lipoyl synthase, mitochondrial | 0.82 | 1.9E-02 |
| Q9XX28 | DeHydrogenases, Short chain | 0.82 | 8.0E-03 |
| Q4W5T0 | Mitochondrial Ribosomal Protein, Large | 0.81 | 3.6E-03 |
| Q9U348 | COLlagen | 0.81 | 7.8E-03 |
| Q9XXN2 | MICOS complex subunit MIC60 | 0.81 | 6.9E-12 |
| RL9 | 60S ribosomal protein L9 | 0.81 | 1.1E-02 |
| A0A061AKV1 | UDP-glucuronosyltransferase | 0.80 | 6.2E-04 |
| Q9N3T5 | SPG (Spastic paraplegia) | 0.80 | 6.1E-03 |
| Q94055 | Serine--pyruvate aminotransferase | 0.80 | 1.1E-06 |
| PAT2 | Integrin alpha pat-2 | 0.79 | 1.4E-02 |
| SODC | Superoxide dismutase [Cu-Zn] | 0.79 | 6.7E-03 |
| O44619 | Carnitine Palmitoyl Transferase | 0.78 | 1.1E-06 |
| O61818 | Mitochondrial Ribosomal Protein, Small | 0.78 | 1.3E-06 |
| DCXR | L-xylulose reductase | 0.78 | 1.5E-02 |
| DIF1 | Protein dif-1 | 0.78 | 3.5E-02 |
| LRP | Low-density lipoprotein receptor-related protein | 0.77 | 9.9E-05 |
| G5ED31 | Ubiquinol-Cytochrome c oxidoReductase complex | 0.77 | 1.0E-03 |
| ODB2 | Lipoamide acyltransferase component of branched-chain alpha-keto acid dehydrogenase complex, mitochondrial | 0.77 | 3.6E-02 |
| RM41 | 39S ribosomal protein L41, mitochondrial | 0.77 | 2.7E-03 |
| TBCB | Tubulin-specific chaperone B | 0.76 | 2.7E-02 |
| Q9XWD1 | Fatty Acid CoA Synthetase family | 0.75 | 2.5E-03 |
| G5ECD0 | CYtochrome P450 family | 0.75 | 2.8E-03 |
| DNJ10 | DnaJ homolog dnj-10 | 0.75 | 1.7E-07 |
| O62107 | UDP-GALactose 4-Epimerase | 0.73 | 1.4E-02 |
| ACD11 | Acyl-CoA dehydrogenase family member 11 | 0.73 | 2.5E-06 |
| CYSK2 | Bifunctional L-3-cyanoalanine synthase/cysteine synthase | 0.72 | 3.8E-03 |
| Q9XTU9 | Glutaredoxin | 0.72 | 3.3E-03 |
| RT09 | Probable 40S ribosomal protein S9, mitochondrial | 0.72 | 4.0E-05 |
| Q9NAG2 | Mitochondrial Ribosomal Protein, Large | 0.72 | 8.8E-04 |
| G5EDQ0 | Chloride channel protein | 0.72 | 4.8E-02 |
| MFRN | Mitoferrin | 0.72 | 2.5E-02 |
| Q19130 | Glutamine-Fructose 6-phosphate AminoTransferase homolog | 0.71 | 7.3E-03 |
| MPC1 | Probable mitochondrial pyruvate carrier 1 | 0.70 | 2.0E-02 |
| NDUS7 | Probable NADH dehydrogenase [ubiquinone] iron-sulfur protein 7, mitochondrial | 0.70 | 7.7E-04 |
| CPR5 | Cathepsin B-like cysteine proteinase 5 | 0.69 | 2.3E-02 |
| UGT47 | Putative UDP-glucuronosyltransferase ugt-47 | 0.69 | 1.5E-04 |
| AGT2L | Alanine--glyoxylate aminotransferase 2-like | 0.69 | 2.6E-02 |
| Q9TYJ8 | Mitochondrial Ribosomal Protein, Large | 0.69 | 2.8E-02 |
| RM45 | Probable 39S ribosomal protein L45, mitochondrial | 0.68 | 2.5E-04 |
| O17612 | Enoyl-CoA Hydratase | 0.68 | 2.5E-03 |
| INX12 | Innexin-12 | 0.68 | 1.3E-02 |
| ODP2 | Dihydrolipoyllysine-residue acetyltransferase component of pyruvate dehydrogenase complex, mitochondrial | 0.67 | 9.1E-03 |
| RT15 | 28S ribosomal protein S15, mitochondrial | 0.67 | 5.2E-05 |
| Q95PY4 | Mitochondrial Ribosomal Protein, Small | 0.66 | 1.3E-05 |
| RM51 | 39S ribosomal protein L51, mitochondrial | 0.66 | 1.0E-02 |
| TPP2 | Tripeptidyl-peptidase 2 | 0.66 | 6.8E-07 |
| GCP | Bifunctional glyoxylate cycle protein | 0.65 | 3.4E-12 |
| P4HA2 | Prolyl 4-hydroxylase subunit alpha-2 | 0.65 | 4.3E-03 |
| O17805 | COLlagen | 0.64 | 4.0E-02 |
| HACD | Very-long-chain (3R)-3-hydroxyacyl-CoA dehydratase hpo-8 | 0.64 | 2.5E-02 |
| GRPE | GrpE protein homolog, mitochondrial | 0.64 | 2.3E-02 |
| Q9BPN6 | Mitochondrial Ribosomal Protein, Large | 0.62 | 2.2E-06 |
| RS4 | 40S ribosomal protein S4 | 0.62 | 1.6E-12 |
| O18000 | Patterned Expression Site | 0.62 | 2.3E-02 |
| UCR1 | Cytochrome b-c1 complex subunit 1, mitochondrial | 0.62 | 7.1E-03 |
| P91306 | C. Elegans Y-box | 0.61 | 4.3E-02 |
| ADAS | Alkyldihydroxyacetonephosphate synthase | 0.61 | 3.3E-02 |
| Q22341 | TransThyretin-Related family domain | 0.61 | 3.5E-02 |
| COX2 | Cytochrome c oxidase subunit 2 | 0.60 | 6.4E-03 |
| G5EG54 | Carnitine Palmitoyl Transferase | 0.60 | 2.6E-02 |
| RT31 | 28S ribosomal protein S31, mitochondrial | 0.59 | 1.0E-04 |
| Q9GZD1 | UDP-GlucuronosylTransferase | 0.59 | 8.5E-03 |
| U562 | UPF0562 protein C29E4.12 | 0.59 | 3.2E-02 |
| Q20062 | Mitochondrial Associated RiBonuclease homolog | 0.59 | 2.7E-03 |
| BST14 | Bestrophin homolog 14 | 0.58 | 1.5E-02 |

B. **Enriched in Day 7 and depleted in Day 4**

| **Protein ID** | **Protein Description** | **AVG.Log2.Ratio** | **Q value** |
| --- | --- | --- | --- |
| P91350 | Nuclear Pore complex Protein | -0.58 | 1.0E-02 |
| RCC1 | Regulator of chromosome condensation | -0.58 | 7.4E-03 |
| CAN | Calpain clp-1 | -0.58 | 7.5E-03 |
| RL17 | 60S ribosomal protein L17 | -0.58 | 6.4E-05 |
| PROF1 | Profilin-1 | -0.59 | 4.0E-06 |
| Q965S5 | SyNapTotagmin | -0.59 | 3.8E-02 |
| RL38 | 60S ribosomal protein L38 | -0.59 | 1.4E-02 |
| G5ED36 | Endocytosis protein RME-8;Receptor Mediated Endocytosis | -0.59 | 9.8E-03 |
| O17766 | Lipase | -0.59 | 5.7E-03 |
| ILKH | Integrin-linked protein kinase homolog pat-4 | -0.60 | 5.0E-03 |
| Q9GPA0 | UDP-Glucose Glycoprotein glucosylTransferase | -0.60 | 1.1E-22 |
| CYP5 | Peptidyl-prolyl cis-trans isomerase 5 | -0.60 | 2.6E-03 |
| GBB1 | Guanine nucleotide-binding protein subunit beta-1 | -0.61 | 3.6E-03 |
| Q9XVS9 | DeHydrogenases, Short chain | -0.62 | 2.2E-02 |
| RLA0 | 60S acidic ribosomal protein P0 | -0.62 | 7.5E-12 |
| SYRC | Probable arginine--tRNA ligase, cytoplasmic;Arginyl(R) Amino-acyl tRNA Synthetase | -0.62 | 1.6E-03 |
| NDX6 | Putative nudix hydrolase 6 | -0.62 | 3.7E-02 |
| RS26 | 40S ribosomal protein S26 | -0.62 | 2.2E-04 |
| SURF4 | Surfeit locus protein 4 homolog | -0.63 | 4.8E-02 |
| Q94230 | Pur alpha Like Protein | -0.63 | 1.2E-07 |
| RL13A | 60S ribosomal protein L13a | -0.64 | 9.8E-07 |
| Q9U2Z1 | Yeast SEC homolog | -0.64 | 2.9E-02 |
| Q5TKA3 | Fatty Acid CoA Synthetase family | -0.64 | 1.7E-03 |
| U520 | Putative U5 small nuclear ribonucleoprotein 200 kDa helicase | -0.64 | 8.3E-11 |
| PSMD3 | 26S proteasome non-ATPase regulatory subunit 3 | -0.65 | 6.4E-04 |
| RPN2 | Dolichyl-diphosphooligosaccharide--protein glycosyltransferase subunit 2 | -0.65 | 1.4E-04 |
| IMP2 | Intramembrane protease 2 | -0.65 | 1.1E-08 |
| Q19371 | Yeast SEC homolog | -0.66 | 1.0E-02 |
| KINH | Kinesin heavy chain | -0.66 | 1.3E-06 |
| YVRI | GILT-like protein F37H8.5 | -0.67 | 1.2E-04 |
| Q9GZH5 | Proteasome Regulatory Particle, Non-ATPase-like | -0.67 | 3.2E-05 |
| O61792 | Proteasome Regulatory Particle, Non-ATPase-like | -0.67 | 8.0E-03 |
| RS13 | 40S ribosomal protein S13 | -0.67 | 4.1E-06 |
| GYS | Glycogen [starch] synthase | -0.67 | 6.7E-03 |
| Q18231 | Ribosomal Protein, Small subunit | -0.67 | 1.0E-03 |
| PHB2 | Mitochondrial prohibitin complex protein 2 | -0.68 | 2.6E-02 |
| VIP1 | Inositol hexakisphosphate and diphosphoinositol-pentakisphosphate kinase | -0.68 | 2.9E-03 |
| H1UBL1 | MItochondrial Calcium Uptake protein;Calcium uptake protein 1 homolog | -0.69 | 4.0E-02 |
| RL23 | 60S ribosomal protein L23 | -0.69 | 7.4E-06 |
| P91495 | Nuclear Pore complex Protein | -0.69 | 1.1E-20 |
| G3MU79 | Ubiquitin carboxyl-terminal hydrolase 7 | -0.69 | 1.2E-03 |
| RS15 | 40S ribosomal protein S15 | -0.70 | 9.7E-06 |
| MOGS1 | Mannosyl-oligosaccharide glucosidase | -0.70 | 1.1E-03 |
| NOC2L | Nucleolar complex protein 2 homolog | -0.71 | 2.9E-03 |
| Q23378 | TransThyretin-Related family domain | -0.71 | 8.8E-03 |
| Q9XWH0 | Yeast BUB homolog | -0.71 | 3.4E-02 |
| ACLY | Probable ATP-citrate synthase | -0.71 | 4.9E-05 |
| RL27 | 60S ribosomal protein L27 | -0.71 | 4.2E-04 |
| ASNA | ATPase asna-1 | -0.72 | 3.0E-04 |
| Q4JFH6 | SaPosin-like Protein family | -0.73 | 2.7E-02 |
| O61977 | TransThyretin-Related family domain | -0.73 | 2.3E-02 |
| Q23588 | Uridine PhosPhorylase | -0.73 | 3.1E-02 |
| RL7A | 60S ribosomal protein L7a | -0.74 | 1.6E-06 |
| IMPA1 | Inositol monophosphatase ttx-7 | -0.74 | 7.8E-05 |
| Q9TZL9 | Fatty Acyl-CoA ReDuctase | -0.75 | 2.6E-02 |
| TCPD | T-complex protein 1 subunit delta | -0.75 | 3.8E-10 |
| GMPR | GMP reductase | -0.75 | 3.9E-03 |
| ISW1 | Chromatin-remodeling complex ATPase chain isw-1 | -0.76 | 3.2E-02 |
| G5ECZ0 | Heavy chain, Unconventional Myosin | -0.76 | 4.7E-02 |
| B3WFV9 | Anion exchange protein | -0.76 | 7.2E-04 |
| EIF3A | Eukaryotic translation initiation factor 3 subunit A | -0.77 | 2.9E-12 |
| RSP2 | Probable splicing factor, arginine/serine-rich 2 | -0.77 | 1.6E-04 |
| Q23368 | Yeast SEC homolog | -0.77 | 5.0E-03 |
| UN104 | Kinesin-like protein unc-104 | -0.77 | 1.2E-05 |
| RL4 | 60S ribosomal protein L4 | -0.77 | 4.6E-19 |
| EIF3B | Eukaryotic translation initiation factor 3 subunit B | -0.77 | 5.2E-08 |
| O62415 | LYSozyme | -0.77 | 5.0E-03 |
| AKAP1 | KH domain-containing protein akap-1 | -0.79 | 4.1E-03 |
| Q9TZD9 | HAlF transporter (PGP related) | -0.79 | 5.8E-06 |
| RL8 | 60S ribosomal protein L8 | -0.79 | 2.5E-13 |
| LDH | L-lactate dehydrogenase | -0.80 | 8.5E-04 |
| PRS10 | Probable 26S protease regulatory subunit 10B | -0.80 | 2.7E-07 |
| NTF2 | Probable nuclear transport factor 2 | -0.80 | 4.9E-02 |
| P92005 | CathePsin Z | -0.80 | 8.6E-11 |
| UNC87 | Protein unc-87 | -0.81 | 1.3E-06 |
| TCPG | T-complex protein 1 subunit gamma | -0.81 | 3.2E-09 |
| EIF3C | Eukaryotic translation initiation factor 3 subunit C | -0.81 | 3.4E-04 |
| Q7YZW5 | VEMA (Mammalian ventral midline antigen) related | -0.81 | 4.3E-02 |
| PHO5 | Putative acid phosphatase 5 | -0.81 | 4.0E-03 |
| Q21750 | Acid Alpha Glucosidase Relate | -0.82 | 1.5E-05 |
| P91027 | Calponin Homology Domain containing Protein | -0.82 | 1.9E-05 |
| G5ECX8 | Increased Sodium Tolerance Related | -0.82 | 2.7E-02 |
| U2AF2 | Splicing factor U2AF 65 kDa subunit;U2AF splicing factor | -0.82 | 6.1E-03 |
| AKT1 | Serine/threonine-protein kinase akt-1 | -0.82 | 1.9E-02 |
| Q21801 | GAlactosidase/N-Acetylgalactosaminidase | -0.82 | 1.0E-05 |
| PLBL1 | Putative phospholipase B-like 1 | -0.82 | 7.4E-18 |
| OLA1 | Obg-like ATPase 1 | -0.83 | 6.0E-06 |
| RS21 | 40S ribosomal protein S21 | -0.83 | 8.9E-10 |
| P91277 | Heterogeneous nuclear RibonucleoProtein (HnRNP) K homolog | -0.83 | 1.1E-03 |
| CPLX1 | Putative complexin-1 | -0.84 | 2.8E-02 |
| RS12 | 40S ribosomal protein S12 | -0.85 | 6.5E-03 |
| Q18886 | Related to yeast Vacuolar Protein Sorting factor | -0.86 | 1.5E-03 |
| Q22170 | Intracellular LEctin | -0.86 | 2.5E-02 |
| SYDC | Aspartate--tRNA ligase, cytoplasmic | -0.86 | 9.0E-05 |
| Q22716 | Ribosomal Protein, Large subunit | -0.87 | 9.2E-03 |
| KS6A2 | Putative ribosomal protein S6 kinase alpha-2 | -0.88 | 1.9E-02 |
| CYP3 | Peptidyl-prolyl cis-trans isomerase 3 | -0.88 | 1.2E-02 |
| O44782 | Major sperm protein | -0.88 | 5.5E-03 |
| Q23382 | CCDC (Human Coiled Coil Domain Containing) homolog | -0.88 | 8.2E-07 |
| CAND1 | Cullin-associated NEDD8-dissociated protein 1 | -0.89 | 6.0E-05 |
| PHM | Probable peptidylglycine alpha-hydroxylating monooxygenase 1 | -0.90 | 1.3E-03 |
| PFD3 | Probable prefoldin subunit 3 | -0.90 | 1.1E-03 |
| O62334 | AGL (Amylo-1,6-GLucosidase, 4-alpha-glucanotransferase) glycogen debranching enzyme | -0.91 | 7.3E-04 |
| FBRL | rRNA 2'-O-methyltransferase fibrillarin | -0.91 | 2.4E-04 |
| Q20306 | ABC transporter, class F | -0.91 | 4.8E-03 |
| SDHF2 | Succinate dehydrogenase assembly factor 2, mitochondrial | -0.91 | 1.3E-02 |
| PROF2 | Profilin-2 | -0.91 | 2.9E-05 |
| G5EF26 | Putative low density lipoprotein receptor associated protein (37.4 kD) | -0.92 | 7.1E-03 |
| H2L0C0 | EMC Endoplasmic Membrane protein Complex (Yeast EMC) homolog | -0.93 | 4.0E-09 |
| PLBL2 | Putative phospholipase B-like 2 | -0.93 | 3.4E-13 |
| COPD | Probable coatomer subunit delta | -0.94 | 3.4E-03 |
| Q20277 | FIP (Fungus-Induced Protein) Related | -0.94 | 1.9E-03 |
| GYG1 | Glycogenin-1 | -0.94 | 1.4E-03 |
| SYSC | Probable serine--tRNA ligase, cytoplasmic | -0.95 | 6.0E-05 |
| DX39B | Spliceosome RNA helicase DDX39B homolog | -0.95 | 6.1E-07 |
| ELO6 | Elongation of very long chain fatty acids protein 6 | -0.96 | 4.7E-02 |
| SYNC | Asparagine--tRNA ligase, cytoplasmic | -0.96 | 1.4E-02 |
| Q86NH9 | TransThyretin-Related family domain | -0.96 | 2.7E-02 |
| Q9TYS8 | CALUmenin (Calcium-binding protein) homolog | -0.96 | 1.3E-03 |
| Q22240 | Ubiquitin carboxyl-terminal hydrolase | -0.96 | 1.8E-02 |
| KAD1 | Adenylate kinase isoenzyme 1 | -0.96 | 2.3E-03 |
| AT131 | Probable manganese-transporting ATPase catp-8 | -0.96 | 2.3E-08 |
| ADF1 | Actin-depolymerizing factor 1, isoforms a/b | -0.97 | 2.0E-02 |
| O44985 | Tumorous Enhancer of Glp-1(Gf) | -0.97 | 2.6E-08 |
| G8JY03 | DEAD boX helicase homolog;Pre-mRNA-splicing factor ATP-dependent RNA helicase ddx-15 | -0.97 | 7.7E-03 |
| A3QMC6 | ERLin (ER lipid raft associated protein) homolog | -0.97 | 4.5E-04 |
| Q21473 | AQuaPorin or aquaglyceroporin related | -0.98 | 2.2E-02 |
| RS7 | 40S ribosomal protein S7 | -0.98 | 3.1E-07 |
| G5EC40 | Trehalase | -0.98 | 4.0E-02 |
| MDHM | Probable malate dehydrogenase, mitochondrial | -0.99 | 1.8E-05 |
| SPCS2 | Probable signal peptidase complex subunit 2 | -0.99 | 5.4E-04 |
| Q21746 | Small Glutamine-rich Tetratrico repeat protein | -0.99 | 1.9E-02 |
| A5HU98 | GUanylate Kinase | -0.99 | 3.4E-05 |
| SC61G | Protein transport protein Sec61 subunit gamma | -1.00 | 5.0E-03 |
| Q22947 | RH (Rhesus) antigen Related | -1.00 | 4.6E-02 |
| IF4A | Eukaryotic initiation factor 4A | -1.00 | 1.1E-03 |
| LARP1 | La-related protein 1 | -1.00 | 6.2E-03 |
| Q20829 | Alpha-mannosidase | -1.00 | 7.0E-06 |
| PGK | Probable phosphoglycerate kinase | -1.01 | 3.1E-07 |
| EIF3L | Eukaryotic translation initiation factor 3 subunit L | -1.01 | 2.9E-04 |
| COPB2 | Probable coatomer subunit beta' | -1.01 | 3.7E-04 |
| G5EC23 | HCF1 related | -1.01 | 4.5E-04 |
| RS17 | 40S ribosomal protein S17 | -1.02 | 2.3E-09 |
| Q965V4 | EXPOrtin (Nuclear export receptor) | -1.02 | 2.6E-02 |
| PRP19 | Pre-mRNA-processing factor 19 | -1.02 | 9.8E-04 |
| YQ4B | Putative pseudouridine synthase B0024.11 | -1.03 | 3.5E-03 |
| RL7 | 60S ribosomal protein L7 | -1.03 | 4.1E-09 |
| RPF2 | Ribosome production factor 2 homolog | -1.04 | 1.7E-02 |
| Q9U1W8 | LSM Sm-like protein | -1.06 | 2.6E-02 |
| PFD4 | Probable prefoldin subunit 4 | -1.07 | 1.2E-02 |
| CNOT7 | CCR4-NOT transcription complex subunit 7 | -1.08 | 9.1E-04 |
| STDH1 | Putative steroid dehydrogenase 1 | -1.08 | 3.7E-02 |
| TORS | Torsin-like protein | -1.08 | 4.7E-04 |
| Q21832 | RNA-binding protein 8A | -1.09 | 1.4E-04 |
| SQV2 | Beta-1,3-galactosyltransferase sqv-2 | -1.09 | 2.5E-02 |
| ADRM1 | Proteasomal ubiquitin receptor ADRM1 homolog | -1.10 | 3.9E-04 |
| PPM1 | Protein phosphatase ppm-1 | -1.10 | 9.5E-03 |
| Q9N363 | mRNA DeCAPping enzyme | -1.10 | 1.1E-02 |
| RL10 | 60S ribosomal protein L10 | -1.11 | 1.8E-08 |
| RPAB3 | Probable DNA-directed RNA polymerases I, II, and III subunit RPABC3 | -1.11 | 7.4E-03 |
| LMP1 | LAMP family protein lmp-1 | -1.11 | 9.1E-05 |
| Q17796 | Hepatocyte Growth factor-Regulated TK Substrate (HRS) family | -1.11 | 4.2E-02 |
| PP2C2 | Probable protein phosphatase 2C T23F11.1 | -1.11 | 2.3E-02 |
| O61742 | Proteasome Regulatory Particle, Non-ATPase-like | -1.12 | 2.0E-02 |
| Q20107 | Peptidyl-prolyl cis-trans isomerase | -1.12 | 1.4E-02 |
| BTF3 | Transcription factor BTF3 homolog | -1.12 | 2.7E-05 |
| TBA3 | Tubulin alpha-3 chain | -1.13 | 6.7E-04 |
| O16482 | CYtochrome P450 family | -1.13 | 3.7E-03 |
| OTUBL | Ubiquitin thioesterase otubain-like | -1.13 | 2.2E-02 |
| O62289 | TransThyretin-Related family domain | -1.13 | 4.5E-02 |
| P90961 | Clathrin LIght Chain | -1.13 | 1.8E-02 |
| Q9N4N4 | SWI/SNF nucleosome remodeling complex component | -1.13 | 6.9E-04 |
| PAR5 | 14-3-3-like protein 1 | -1.14 | 2.2E-04 |
| Q27492 | DNA-directed RNA polymerase subunit beta | -1.14 | 4.2E-02 |
| RS5 | 40S ribosomal protein S5 | -1.15 | 3.6E-06 |
| MMSA | Probable methylmalonate-semialdehyde dehydrogenase [acylating], mitochondrial | -1.15 | 2.4E-03 |
| Q1XFY9 | Ribosomal Protein, Small subunit | -1.15 | 1.5E-08 |
| G5EFP5 | Alpha1,3-fucosyltransferase homologue | -1.16 | 7.3E-03 |
| Q9XWP7 | Eukaryotic Initiation Factor | -1.16 | 1.1E-02 |
| UBP14 | Ubiquitin carboxyl-terminal hydrolase 14 | -1.16 | 8.6E-04 |
| RL37A | 60S ribosomal protein L37a | -1.16 | 5.6E-03 |
| MTAP | S-methyl-5'-thioadenosine phosphorylase | -1.17 | 2.8E-02 |
| STT3 | Dolichyl-diphosphooligosaccharide--protein glycosyltransferase subunit stt-3 | -1.17 | 3.5E-03 |
| G5EGS9 | N-acetyllactosamine synthase | -1.19 | 5.7E-04 |
| Q9N3C9 | RNA Polymerase II (B) subunit | -1.20 | 2.3E-02 |
| Q9U2S6 | Peptidyl-prolyl cis-trans isomerase E | -1.20 | 1.3E-02 |
| TCPZ | T-complex protein 1 subunit zeta | -1.20 | 3.1E-03 |
| HSP7C | Heat shock 70 kDa protein C | -1.20 | 8.5E-09 |
| Q93878 | Yeast ERV (ER to Golgi transport Vesicle protein) homolog | -1.22 | 1.1E-02 |
| Q9XTT9 | Proteasome Regulatory Particle, ATPase-like | -1.22 | 7.6E-03 |
| LSM4 | Probable U6 snRNA-associated Sm-like protein LSm4 | -1.22 | 1.1E-02 |
| DMON2 | DOMON domain-containing protein Y73F4A.1 | -1.22 | 1.9E-05 |
| UNC84 | Nuclear migration and anchoring protein unc-84 | -1.23 | 9.5E-04 |
| Q21559 | Ref/ALY RNA export adaptor family | -1.23 | 7.3E-05 |
| CYP6 | Peptidyl-prolyl cis-trans isomerase 6 | -1.23 | 2.7E-06 |
| AAPK2 | 5'-AMP-activated protein kinase catalytic subunit alpha-2 | -1.25 | 8.4E-04 |
| H2KZJ9 | Galectin | -1.25 | 1.5E-02 |
| A5Z2S4 | SAPS (Phosphatase associated) domain protein | -1.25 | 2.2E-02 |
| Q21351 | Ras-Gtpase-activating protein SH3 (Three) domain-Binding Protein | -1.25 | 3.6E-08 |
| P91502 | Ubiquitin carboxyl-terminal hydrolase | -1.25 | 1.7E-04 |
| TECR | Probable very-long-chain enoyl-CoA reductase art-1 | -1.26 | 1.9E-09 |
| RCANL | Calcipressin-like protein | -1.26 | 2.2E-03 |
| Q27526 | Beta-galactosidase | -1.27 | 5.5E-03 |
| NACA | Nascent polypeptide-associated complex subunit alpha | -1.27 | 1.9E-02 |
| TPSTA | Protein-tyrosine sulfotransferase A | -1.27 | 1.6E-02 |
| IF5A2 | Eukaryotic translation initiation factor 5A-2 | -1.28 | 9.6E-03 |
| TCPB | T-complex protein 1 subunit beta | -1.28 | 1.2E-13 |
| ADR2 | Probable double-stranded RNA-specific adenosine deaminase | -1.28 | 7.4E-03 |
| RSP1 | Probable splicing factor, arginine/serine-rich 1 | -1.28 | 6.1E-05 |
| Q21831 | SNF chromatin remodeling Complex component | -1.32 | 3.6E-03 |
| H2KZJ5 | GLutaRedoXin | -1.33 | 2.9E-02 |
| EIF3H | Eukaryotic translation initiation factor 3 subunit H | -1.33 | 7.4E-06 |
| RUVB2 | RuvB-like 2 | -1.33 | 7.0E-05 |
| PSA2 | Proteasome subunit alpha type-2 | -1.34 | 1.3E-04 |
| Q9NLD1 | HnRNP A1 homolog | -1.34 | 1.2E-09 |
| GRP1 | GTP exchange factor for ARFs 1 | -1.35 | 2.5E-02 |
| Q20752 | STCH (Truncated HSP) family | -1.35 | 5.3E-05 |
| Q94207 | SUPpressor | -1.36 | 2.5E-02 |
| IF2B | Eukaryotic translation initiation factor 2 subunit 2 | -1.37 | 2.3E-03 |
| ALF2 | Fructose-bisphosphate aldolase 2 | -1.38 | 5.0E-02 |
| G5ECL3 | Pre-RNA processing 21 | -1.38 | 8.9E-03 |
| G5EE04 | Hsp-70 Interacting Protein homolog | -1.39 | 5.4E-04 |
| Q9U2F6 | Related to yeast Vacuolar Protein Sorting factor | -1.39 | 1.2E-02 |
| H2KYR1 | VIG (Drosophila Vasa Intronic Gene) ortholog | -1.39 | 2.3E-07 |
| ARL3 | ADP-ribosylation factor-like protein 3 | -1.39 | 5.1E-06 |
| G1K108 | Amino acid Transporter GlycoProtein subunit | -1.40 | 1.5E-04 |
| Q8IA75 | Phosphoinositide phospholipase C | -1.40 | 4.5E-02 |
| Q93565 | ToLlIp homolog | -1.40 | 2.1E-02 |
| HEXA | Beta-hexosaminidase A | -1.40 | 1.8E-09 |
| Q9XXA2 | Microtubule End Binding Protein | -1.40 | 4.1E-02 |
| O16720 | Protein argonaute | -1.42 | 1.9E-02 |
| O76371 | Proteasome Regulatory Particle, ATPase-like | -1.43 | 2.9E-05 |
| RPB2 | DNA-directed RNA polymerase II subunit RPB2 | -1.43 | 7.2E-05 |
| G5EBQ9 | SIPA (Vertebrate Signal-Induced Proliferation-Associated) homolog | -1.43 | 2.0E-03 |
| PDI2 | Protein disulfide-isomerase 2 | -1.43 | 2.2E-02 |
| O44729 | Yeast PRP (Splicing factor) related | -1.44 | 3.5E-03 |
| G5EES3 | Protein argonaute | -1.45 | 8.2E-09 |
| P90787 | alpha-1,2-Mannosidase | -1.47 | 1.6E-04 |
| H2KZK7 | Dipeptidyl Peptidase Four (IV) family | -1.47 | 1.0E-03 |
| RS23 | 40S ribosomal protein S23 | -1.47 | 1.5E-06 |
| ADPGK | Probable ADP-dependent glucokinase | -1.47 | 2.5E-07 |
| DAF31 | N-alpha-acetyltransferase daf-31 | -1.47 | 4.0E-02 |
| YQ83 | GYF domain-containing protein C18H9.3 | -1.47 | 1.8E-03 |
| Q9TXU7 | Eukaryotic Initiation Factor | -1.48 | 3.3E-02 |
| Q21152 | Fatty Acid/Retinol binding protein | -1.48 | 1.3E-02 |
| RL21 | 60S ribosomal protein L21 | -1.49 | 3.5E-06 |
| Q9U1V9 | DNaJ domain (Prokaryotic heat shock protein) | -1.49 | 6.6E-05 |
| O17406 | AT hook Transcription Factor family | -1.51 | 2.3E-07 |
| K8ERX1 | Zipcode Binding Protein homolog | -1.51 | 1.1E-02 |
| TMED2 | Suppressor/enhancer of lin-12 protein 9 | -1.53 | 2.7E-03 |
| A3QMC5 | Ribosomal Protein, Large subunit | -1.53 | 5.7E-06 |
| SRP19 | Probable signal recognition particle 19 kDa protein | -1.53 | 4.1E-03 |
| Q95002 | Serine/threonine-protein phosphatase | -1.53 | 1.3E-03 |
| LI15B | Protein lin-15B | -1.54 | 2.8E-07 |
| PFD1 | Probable prefoldin subunit 1 | -1.54 | 4.5E-02 |
| Q23463 | Elongation Factor TU family | -1.54 | 3.7E-10 |
| G5EDL7 | RILP (Rab7-Interacting Lysosomal Protein) homolog | -1.54 | 2.7E-02 |
| Q19440 | Phosphotransferase | -1.54 | 3.0E-04 |
| Q20684 | GaLECtin | -1.55 | 9.6E-05 |
| TOP2 | Probable DNA topoisomerase 2 | -1.56 | 5.7E-12 |
| Q21551 | Coiled coil Helix Coiled coiled Helix domain | -1.56 | 3.7E-02 |
| Q19162 | Ribosomal Protein, Large subunit | -1.56 | 4.3E-02 |
| RS19 | 40S ribosomal protein S19 | -1.57 | 8.0E-07 |
| GSLG1 | Golgi apparatus protein 1 homolog | -1.58 | 1.2E-11 |
| G5ED46 | CAlSYntenin/Alcadein homolog | -1.58 | 1.1E-02 |
| DAD1 | Dolichyl-diphosphooligosaccharide--protein glycosyltransferase subunit dad-1 | -1.59 | 4.2E-02 |
| Q9U1X6 | EMC Endoplasmic Membrane protein Complex (Yeast EMC) homolog | -1.59 | 1.1E-03 |
| Q22743 | RAs-related GTP-binding protein A | -1.59 | 2.5E-03 |
| O76630 | EMC Endoplasmic Membrane protein Complex (Yeast EMC) homolog | -1.59 | 1.3E-08 |
| Q9XVE9 | Ribosomal Protein, Large subunit | -1.62 | 3.6E-07 |
| LEM2 | LEM protein 2 | -1.62 | 9.3E-05 |
| G5ED01 | INOsitol-3-phosphate Synthase | -1.63 | 3.1E-11 |
| Q967F1 | Eukaryotic Initiation Factor | -1.64 | 2.7E-02 |
| Q9GUP2 | Enhancer of EfL-1 mutant phenotype | -1.64 | 3.0E-02 |
| SMD3 | Small nuclear ribonucleoprotein Sm D3 | -1.64 | 9.6E-06 |
| RUXG | Probable small nuclear ribonucleoprotein G | -1.65 | 1.6E-03 |
| RL36 | 60S ribosomal protein L36 | -1.65 | 7.6E-07 |
| HDA1 | Histone deacetylase 1 | -1.65 | 1.7E-06 |
| PRP8 | Pre-mRNA-splicing factor 8 homolog | -1.65 | 3.7E-13 |
| Q20774 | DNaJ domain (Prokaryotic heat shock protein) | -1.66 | 6.6E-03 |
| Q9XW17 | Cytokinesis, Apoptosis, RNA-associated | -1.66 | 6.8E-03 |
| WDR51 | WD repeat-containing protein wdr-5.1 | -1.68 | 1.3E-03 |
| YLC1 | ER membrane protein complex subunit 7 homolog | -1.68 | 6.1E-04 |
| G5EDQ2 | HMG | -1.68 | 1.1E-03 |
| G5EFS2 | MuSashI (Fly neural) family | -1.71 | 3.5E-04 |
| Q21295 | Nuclear Pore complex Protein | -1.72 | 6.8E-03 |
| Q9U2F5 | TIA-1/TIAL RNA binding protein homolog | -1.73 | 1.6E-02 |
| PLDL | Probable phospholipase D F09G2.8 | -1.75 | 8.8E-04 |
| VMP1 | Ectopic P granules protein 3 | -1.77 | 2.1E-02 |
| SRRT | Serrate RNA effector molecule homolog | -1.77 | 4.0E-04 |
| Q8WTJ4 | UBX-containing protein in Nematodes | -1.77 | 1.5E-02 |
| CYC21 | Cytochrome c 2.1 | -1.78 | 2.9E-06 |
| EIF3I | Eukaryotic translation initiation factor 3 subunit I | -1.81 | 2.5E-04 |
| SPT5H | Transcription elongation factor SPT5 | -1.82 | 2.4E-02 |
| NPL11 | Neprilysin-11 | -1.83 | 1.1E-06 |
| O17373 | Intestinal acid PHOsphatase | -1.84 | 2.5E-06 |
| G5EDT9 | UBR E3 ubiquitin ligase homolog | -1.84 | 6.9E-03 |
| RSP6 | Probable splicing factor, arginine/serine-rich 6 | -1.85 | 1.2E-03 |
| O17218 | Ribosomal Protein, Small subunit | -1.87 | 1.7E-05 |
| O62213 | C. Elegans Y-box | -1.87 | 4.3E-06 |
| G5EFE7 | Alpha-(1,6)-fucosyltransferase | -1.87 | 2.7E-03 |
| MGN | Protein mago nashi homolog | -1.87 | 5.6E-05 |
| PPT1 | Palmitoyl-protein thioesterase 1 | -1.88 | 6.4E-03 |
| CEC4 | Chromo domain-containing protein cec-4 | -1.89 | 1.4E-02 |
| SMC3 | Structural maintenance of chromosomes protein 3 | -1.92 | 2.5E-05 |
| Q21945 | Glutathione S-Transferase | -1.93 | 3.5E-03 |
| RS8 | 40S ribosomal protein S8 | -1.93 | 5.9E-09 |
| TACC1 | Transforming acid coiled-coil-containing protein 1 | -1.93 | 1.4E-02 |
| LMN1 | Lamin-1 | -1.94 | 4.5E-22 |
| MEC6 | Mechanosensory abnormality protein 6 | -1.94 | 1.4E-02 |
| Q9XTV4 | MBF (Multiprotein bridging factor) transcriptional coactivator | -1.95 | 3.4E-03 |
| RAB6B | Ras-related protein Rab-6.2 | -1.96 | 4.0E-04 |
| ROA1 | Heterogeneous nuclear ribonucleoprotein A1 | -1.96 | 1.6E-09 |
| PSB3 | Proteasome subunit beta type-3 | -1.96 | 1.7E-02 |
| H12 | Histone H1.2 | -1.96 | 2.1E-07 |
| RU2A | Probable U2 small nuclear ribonucleoprotein A' | -1.98 | 6.1E-06 |
| TNKS1 | Tankyrase-like protein | -1.98 | 4.2E-07 |
| CLAT | Choline O-acetyltransferase | -1.98 | 2.7E-10 |
| RL35 | 60S ribosomal protein L35 | -1.98 | 1.6E-05 |
| Q93641 | SAC1 PIP phosphatase (Yeast Suppressor of ACtin) homolog | -2.00 | 1.4E-03 |
| EF1B1 | Probable elongation factor 1-beta/1-delta 1 | -2.00 | 4.0E-02 |
| G5ED26 | MEL-46 | -2.03 | 1.8E-02 |
| O02325 | PRotein arginine MethylTransferase | -2.05 | 2.9E-02 |
| TCPQ | T-complex protein 1 subunit theta | -2.05 | 2.9E-03 |
| P90949 | Intestinal acid PHOsphatase | -2.06 | 8.3E-04 |
| RS27A | Ubiquitin-like protein 1-40S ribosomal protein S27a | -2.06 | 4.2E-04 |
| MEK2 | Dual specificity mitogen-activated protein kinase kinase mek-2 | -2.08 | 1.8E-02 |
| J7S164 | Histone H2A | -2.12 | 9.6E-09 |
| Q17832 | ViGiLN homolog | -2.13 | 6.6E-03 |
| Q17740 | ALG-1 INteracting protein | -2.13 | 2.2E-05 |
| Q19579 | Polyadenylate-binding protein | -2.13 | 4.3E-03 |
| H13 | Histone H1.3 | -2.15 | 1.5E-02 |
| SRP72 | Signal recognition particle subunit SRP72 | -2.15 | 1.3E-04 |
| C0KDV0 | K+/Cl-Cotransporter | -2.16 | 5.7E-03 |
| H11 | Histone H1.1 | -2.18 | 3.4E-10 |
| SMC1 | Structural maintenance of chromosomes protein 1 | -2.19 | 3.1E-04 |
| ATAT1 | Alpha-tubulin N-acetyltransferase 1 | -2.21 | 3.4E-04 |
| RL24 | 60S ribosomal protein L24 | -2.25 | 7.0E-08 |
| G5EFC4 | Cell Division Cycle related | -2.27 | 2.1E-02 |
| G5EDV3 | C. Elegans Y-box | -2.28 | 1.8E-04 |
| DAF7 | Dauer larva development regulatory growth factor daf-7 | -2.30 | 1.8E-04 |
| SCC3 | Cohesin subunit scc-3 | -2.30 | 2.6E-02 |
| Q9U2M1 | Condensin complex subunit 1 | -2.34 | 1.1E-02 |
| G5EFV4 | HMG | -2.34 | 5.2E-09 |
| CMK1 | Calcium/calmodulin-dependent protein kinase type 1 | -2.40 | 3.1E-02 |
| TPPP | Tubulin polymerization-promoting protein homolog | -2.42 | 2.5E-06 |
| TBB1 | Tubulin beta-1 chain | -2.46 | 1.7E-04 |
| SAE2 | SUMO-activating enzyme subunit uba-2 | -2.49 | 2.0E-02 |
| RS28 | 40S ribosomal protein S28 | -2.55 | 9.0E-07 |
| Q21793 | Prion-like-(Q/N-rich)-domain-bearing protein | -2.56 | 8.7E-05 |
| RS25 | 40S ribosomal protein S25 | -2.56 | 2.0E-05 |
| UFSP | Ufm1-specific protease | -2.59 | 4.5E-09 |
| RL22 | 60S ribosomal protein L22 | -2.64 | 4.1E-07 |
| CDC37 | Probable Hsp90 co-chaperone cdc37;Cell Division Cycle related | -2.66 | 1.6E-02 |
| RL36A | Ribosomal protein L36.A | -2.70 | 2.9E-03 |
| H2AV | Histone H2A.V | -2.73 | 2.8E-04 |
| Q8WQA8 | Ribosomal Protein, Small subunit | -2.73 | 7.6E-06 |
| RS6 | 40S ribosomal protein S6 | -2.75 | 2.3E-05 |
| G5EEL9 | HMG | -2.79 | 2.4E-05 |
| Q22622 | Major sperm protein | -2.84 | 3.3E-03 |
| Q9XWK3 | Nuclear Pore complex Protein | -2.90 | 6.3E-03 |
| RUVB1 | RuvB-like 1 | -2.94 | 1.9E-04 |
| AN321 | Acidic leucine-rich nuclear phosphoprotein 32-related protein 1 | -2.95 | 2.4E-02 |
| Q95QQ1 | DNaJ domain (Prokaryotic heat shock protein) | -3.00 | 3.7E-02 |
| Q17561 | Ref/ALY RNA export adaptor family | -3.02 | 4.1E-04 |
| Q9BIB7 | HnRNP F homolog | -3.10 | 7.4E-03 |
| RSP3 | Probable splicing factor, arginine/serine-rich 3 | -3.13 | 3.8E-09 |
| PP2C1 | Probable protein phosphatase 2C F42G9.1;Uncharacterized protein | -3.14 | 4.0E-03 |
| H4 | Histone H4 | -3.36 | 9.7E-19 |
| TOP1 | DNA topoisomerase 1 | -3.42 | 5.5E-05 |
| Q9UAV3 | Ubiquitin carboxyl-terminal hydrolase | -3.49 | 4.1E-04 |
| RIC3 | Resistance to inhibitors of cholinesterase protein 3 | -3.66 | 5.2E-04 |
| RL28 | 60S ribosomal protein L28 | -3.76 | 5.0E-09 |
| CNEP1 | CTD nuclear envelope phosphatase 1 homolog | -3.89 | 2.9E-02 |
| TM2D4 | TM2 domain-containing protein ZK858.5 | -3.96 | 1.1E-02 |
